# Bis(μ-4-formyl­benzoato-κ^2^ *O*:*O*′)bis­[(4-formyl­benzoato-κ^2^ *O*,*O*′)bis­(iso­nicotin­amide-κ*N* ^1^)copper(II)]

**DOI:** 10.1107/S1600536813010908

**Published:** 2013-04-27

**Authors:** Mustafa Sertçelik, Nagihan Çaylak Delibaş, Hacali Necefoğlu, Tuncer Hökelek

**Affiliations:** a Department of Chemistry, Kafkas University, 63100 Kars, Turkey; b Department of Physics, Sakarya University, 54187 Esentepe, Sakarya, Turkey; c Department of Physics, Hacettepe University, 06800 Beytepe, Ankara, Turkey

## Abstract

The asymmetric unit of the centrosymmetric dinuclear title compound, [Cu_2_(C_8_H_5_O_3_)_4_(C_6_H_6_N_2_O)_4_], contains one half of the complex mol­ecule. The Cu^II^ atoms are bridged by the carboxyl­ate groups of two 4-formyl­benzoate (FOB) anions. Besides the two bridging FOB anions, one additional chelating FOB anion and two isonicotinamide (INA) ligands complete the distorted CuN_2_O_4_ octa­hedral coordination of each Cu^2+^ cation. Within the asymmetric unit, the benzene and pyridine rings are oriented at dihedral angles of 25.1 (3) and 12.6 (3)°, respectively. In the crystal, N—H⋯O and C—H⋯O hydrogen bonds link the mol­ecules into a three-dimensional network. π–π contacts between the pyridine rings [shortest centroid–centroid distance = 3.821 (3) Å] may further stabilize the crystal structure. One of the formyl groups of the two FOB anions is disordered over two sets of sites with an occupancy ratio of 0.65:0.35.

## Related literature

For general background, see: Bigoli *et al.* (1972 ▶); Krishnamachari (1974 ▶). For related structures, see: Hökelek (1996 ▶, 2009*a* ▶,*b* ▶,*c* ▶)); Greenaway *et al.* (1984 ▶); Necefoğlu *et al.* (2011 ▶).
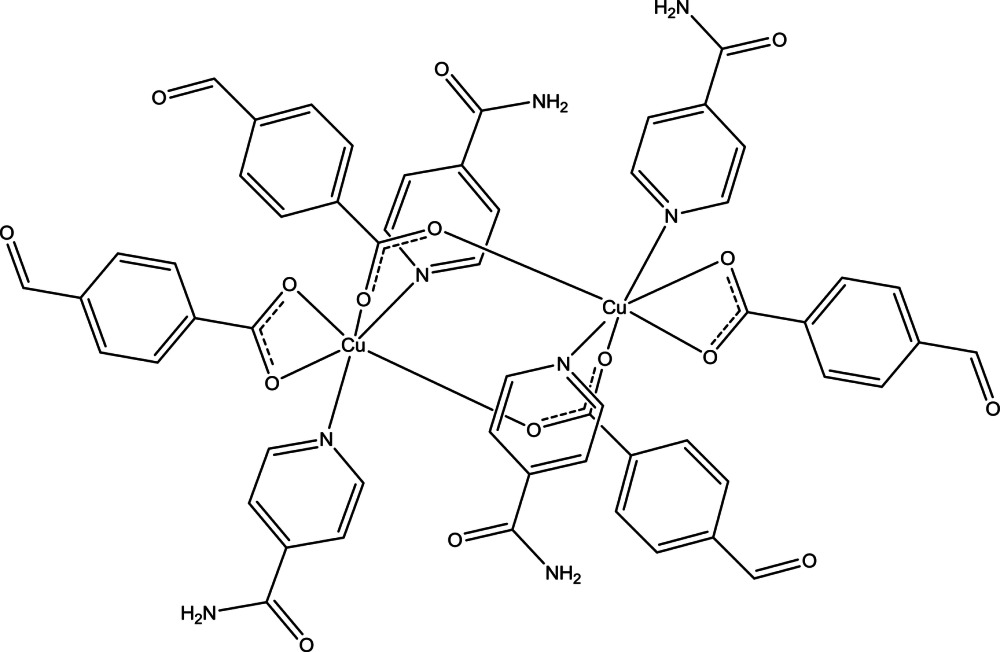


## Experimental

### 

#### Crystal data

[Cu_2_(C_8_H_5_O_3_)_4_(C_6_H_6_N_2_O)_4_]
*M* *_r_* = 1212.09
Triclinic, 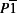

*a* = 8.6462 (2) Å
*b* = 11.6709 (3) Å
*c* = 13.4339 (4) Å
α = 87.876 (3)°
β = 83.483 (3)°
γ = 74.566 (2)°
*V* = 1298.24 (6) Å^3^
*Z* = 1
Mo *K*α radiation
μ = 0.90 mm^−1^
*T* = 100 K
0.17 × 0.07 × 0.06 mm

#### Data collection

Bruker Kappa APEXII CCD area-detector diffractometer
Absorption correction: multi-scan (*SADABS*; Bruker, 2005 ▶) *T*_min_ = 0.927, *T*_max_ = 0.947
19200 measured reflections
6198 independent reflections
4412 reflections with *I* > 2σ(*I*)
*R*_int_ = 0.189

#### Refinement

*R*[*F* ^2^ > 2σ(*F* ^2^)] = 0.078
*wR*(*F* ^2^) = 0.204
*S* = 1.11
6198 reflections
389 parameters
119 restraints
H atoms treated by a mixture of independent and constrained refinement
Δρ_max_ = 1.05 e Å^−3^
Δρ_min_ = −1.51 e Å^−3^

### 

Data collection: *APEX2* (Bruker, 2007 ▶); cell refinement: *SAINT* (Bruker, 2007 ▶); data reduction: *SAINT*; program(s) used to solve structure: *SHELXS97* (Sheldrick, 2008 ▶); program(s) used to refine structure: *SHELXL97* (Sheldrick, 2008 ▶); molecular graphics: *ORTEP-3 for Windows* (Farrugia, 2012 ▶); software used to prepare material for publication: *WinGX* (Farrugia, 2012 ▶) and *PLATON* (Spek, 2009 ▶).

## Supplementary Material

Crystal structure: contains datablock(s) I, global. DOI: 10.1107/S1600536813010908/wm2735sup1.cif

Structure factors: contains datablock(s) I. DOI: 10.1107/S1600536813010908/wm2735Isup2.hkl

Additional supplementary materials:  crystallographic information; 3D view; checkCIF report

## Figures and Tables

**Table 1:** Selected bond lengths (Å)

Cu1—O1	1.994 (3)
Cu1—O2	2.736 (4)
Cu1—O3	1.949 (4)
Cu1—O4^i^	2.242 (3)
Cu1—N1	2.033 (4)
Cu1—N2	2.013 (4)

Symmetry code: (i) 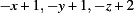.

**Table 2:** Hydrogen-bond geometry (Å, °)

*D*—H⋯*A*	*D*—H	H⋯*A*	*D*⋯*A*	*D*—H⋯*A*
N3—H3*A*⋯O2^ii^	0.86	2.17	2.997 (6)	160
N3—H3*B*⋯O5*A* ^iii^	0.86	2.27	3.044 (9)	149
N4—H4*A*⋯O7^iv^	0.86	2.06	2.878 (6)	158
N4—H4*B*⋯O2^v^	0.86	2.10	2.890 (6)	152
C3—H3⋯O7^vi^	0.93	2.52	3.391 (7)	155
C6—H6⋯O8^vii^	0.93	2.44	3.336 (9)	162
C18—H18⋯O5*A* ^iii^	0.93	2.35	3.274 (9)	169
C23—H23⋯O2^v^	0.93	2.54	3.432 (7)	162
C24—H24⋯O6^viii^	0.93	2.50	3.217 (7)	134

Symmetry codes: (ii) 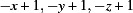; (iii) 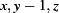; (iv) 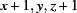; (v) 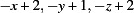; (vi) 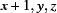; (vii) 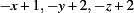; (viii) 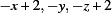.

**Fig. 1.**
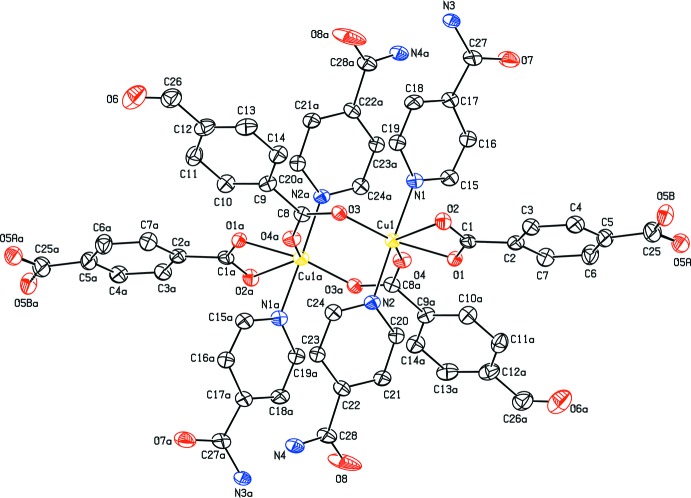
The molecular structure of the title molecule with the atom-numbering scheme. Displacement ellipsoids are drawn at the 50% probability level. Hydrogen atoms have been omitted for clarity [symmetry operator: (a) - x, - y, - z].

## supplementary crystallographic information

### Comment

As a part of our ongoing investigation on transition metal complexes of nicotinamide (NA), one form of niacin (Krishnamachari, 1974), and/or the nicotinic acid derivative *N,N*-diethylnicotinamide (DENA), an important respiratory stimulant (Bigoli *et al.*, 1972), the title compound , [Cu_2_(C_8_H_5_O_3_)_4_(C_6_H_6_N_2_O)_4_], was synthesized and its crystal structure is reported herein.

The asymmetric unit of the centrosymmetric dinuclear title compound contains one half of the complex molecule. The structures of some DENA and/or NA complexes with Zn^II^, *viz*. [Zn_2_(C_11_H_14_NO_2_)_4_(C_10_H_14_N_2_O)_2_] (Hökelek *et al.*, 2009*a*), [Zn_2_(C_8_H_8_NO_2_)_4_(C_10_H_14_N_2_O)_2_]^.^2H_2_O (Hökelek *et al.*, 2009*b*) and [Zn_2_(C_7_H_4_FO_2_)_4_(C_6_H_6_N_2_O)_2_].C_7_H_5_FO_2_ (Hökelek *et al.*, 2009*c*) have also been determined.

In the title dinuclear compound the Cu^2+^ cations are bridged by two carboxylate groups of two 4-formylbenzoate (FOB) anions. The two bridging FOB anions, one chelating FOB anion and two isonicotinamide (INA) ligands coordinate to each Cu^2+^ cation in a distorted octahedral geometry. Each Cu^II^ atom is surrounded by three FOB anions and two INA ligands. The INA ligands are coordinated to the Cu^II^ ion through pyridine N atoms only. Two FOB anions act as bridging ligands, while the other FOB anion is coordinated to the Cu^II^ ion bidentately. The Cu1···Cu1a distance is 4.1554 (8) Å. The four O atoms around the Cu1 atom form a distorted square-planar arrangement with an average Cu1—O bond length of 2.23 Å (Table 1). The distorted octahedral coordination is completed by the pyridine atoms, N1 and N2 of the INA ligands at distances of 2.033 (4) and 2.013 (4) Å, respectively (Table 1, Fig. 1). The N1—Cu1···Cu1*a* and N2—Cu1···Cu1*a* angles (*a* = *x*, - *y*, - *z*) are 97.62 (12) and 77.27 (12) °, respectively. The dihedral angles between the planar carboxylate groups (O1/O2/C1), (O3/O4/C8) and the adjacent benzene rings A (C2—C7), B (C9—C14) are 11.3 (6)° and 2.1 (6)°, respectively, while that between rings A and B is A/B = 25.1 (3)°. The pyridine rings C (N1/C15—C19) and D (N2/C20—C24) are oriented at a dihedral angle of 12.6 (3)°. The O1—Cu1—O2 angle involving the chelating FOB anion is 53.50 (14)°.

The corresponding O—Cu—O angles are 57.75 (2)° in [Cu(C_7_H_4_FO_2_)_2_(C_7_H_5_FO_2_)(C_6_H_6_N_2_O)_2_] (Necefoğlu *et al.*, 2011), 58.3 (3)° in [Cu(C_7_H_5_O_2_)_2_(C_10_H_14_N_2_O)_2_] (Hökelek *et al.*, 1996) and 55.2 (1)° in [Cu(Asp)_2_(py)_2_] (where Asp is acetylsalicylate and py is pyridine) (Greenaway *et al.*, 1984).

In the crystal structure, N—H···O and C–H···O hydrogen bonds (Table 2) link the molecules into a three-dimensional network, in which they may be effective in the stabilization of the structure. The shortest π–π contact between the pyridine rings, *Cg*3—*Cg*4^i^ [symmetry code: (i) 1 - *x*, 1 - *y*, - *z*, where *Cg*3 and *Cg*4 are the centroids of the rings C (N1/C15—C19) and D (N2/C20—C24), respectively] may further stabilize the structure, with a centroid-centroid distance of 3.821 (3) Å.

### Experimental

The title compound was prepared by the reaction of CuSO_4_^.^5H_2_O (1.25 g, 5 mmol) in H_2_O (50 ml) and INA (1.22 g, 10 mmol) in H_2_O (100 ml) with sodium 4-formylbenzoate (1.72 g, 10 mmol) in H_2_O (100 ml). The mixture was filtered and set aside to crystallize at ambient temperature for several days, giving blue single crystals.

### Refinement

The crystal quality of the obtained crystals was poor. Recrystallization studies in order to get a high quality crystal were not successful. Atom H26 (of one formyl group) was located in a difference Fourier map and refined freely. The remaining H atoms were positioned geometrically with N—H = 0.86%A for NH_2_ and C—H = 0.93 Å for aromatic H atoms, and constrained to ride on their parent atoms, with *U*_iso_(H) = 1.2 × *U*_eq_(C,*N*). The other formyl group was found to be disordered over two sets of sites with an occupancy ratio of 0.65:0.35 for O5A, H25A and O5B, H25B. The highest residual electron density was found 0.71 Å from H26 and the deepest hole 0.84 Å from Cu1.

### Crystal data

**Table:**

[Cu_2_(C_8_H_5_O_3_)_4_(C_6_H_6_N_2_O)_4_]	*Z* = 1
*M**_r_* = 1212.09	*F*(000) = 622
Triclinic, *P*1	*D*_x_ = 1.550 Mg m^−^^3^
Hall symbol: -P 1	Mo *K*α radiation, λ = 0.71073 Å
*a* = 8.6462 (2) Å	Cell parameters from 6407 reflections
*b* = 11.6709 (3) Å	θ = 2.4–27.8°
*c* = 13.4339 (4) Å	µ = 0.90 mm^−^^1^
α = 87.876 (3)°	*T* = 100 K
β = 83.483 (3)°	Rod, blue
γ = 74.566 (2)°	0.17 × 0.07 × 0.06 mm
*V* = 1298.24 (6) Å^3^	

### Data collection

**Table:**

Bruker Kappa APEXII CCD area-detector diffractometer	6198 independent reflections
Radiation source: fine-focus sealed tube	4412 reflections with *I* > 2σ(*I*)
Graphite monochromator	*R*_int_ = 0.189
φ and ω scans	θ_max_ = 28.2°, θ_min_ = 1.5°
Absorption correction: multi-scan (*SADABS*; Bruker, 2005)	*h* = −11→11
*T*_min_ = 0.927, *T*_max_ = 0.947	*k* = −15→15
19200 measured reflections	*l* = −16→17

### Refinement

**Table:**

Refinement on *F*^2^	Primary atom site location: structure-invariant direct methods
Least-squares matrix: full	Secondary atom site location: difference Fourier map
*R*[*F*^2^ > 2σ(*F*^2^)] = 0.078	Hydrogen site location: inferred from neighbouring sites
*wR*(*F*^2^) = 0.204	H atoms treated by a mixture of independent and constrained refinement
*S* = 1.11	*w* = 1/[σ^2^(*F*_o_^2^) + (0.076*P*)^2^ + 1.153*P*] where *P* = (*F*_o_^2^ + 2*F*_c_^2^)/3
6198 reflections	(Δ/σ)_max_ < 0.001
389 parameters	Δρ_max_ = 1.05 e Å^−^^3^
119 restraints	Δρ_min_ = −1.51 e Å^−^^3^

### Special details

**Table:**

Geometry. All e.s.d.'s (except the e.s.d. in the dihedral angle between two l.s. planes) are estimated using the full covariance matrix. The cell e.s.d.'s are taken into account individually in the estimation of e.s.d.'s in distances, angles and torsion angles; correlations between e.s.d.'s in cell parameters are only used when they are defined by crystal symmetry. An approximate (isotropic) treatment of cell e.s.d.'s is used for estimating e.s.d.'s involving l.s. planes.
Refinement. Refinement of *F*^2^ against ALL reflections. The weighted *R*-factor *wR* and goodness of fit *S* are based on *F*^2^, conventional *R*-factors *R* are based on *F*, with *F* set to zero for negative *F*^2^. The threshold expression of *F*^2^ > σ(*F*^2^) is used only for calculating *R*-factors(gt) *etc*. and is not relevant to the choice of reflections for refinement. *R*-factors based on *F*^2^ are statistically about twice as large as those based on *F*, and *R*- factors based on ALL data will be even larger.

### Fractional atomic coordinates and isotropic or equivalent isotropic displacement parameters (Å^2^)

**Table:**

	*x*	*y*	*z*	*U*_iso_*/*U*_eq_	Occ. (<1)
Cu1	0.62806 (7)	0.56954 (5)	0.88371 (4)	0.0185 (2)	
O1	0.6218 (4)	0.7313 (3)	0.8280 (2)	0.0219 (8)	
O2	0.8235 (4)	0.6237 (3)	0.7243 (3)	0.0242 (8)	
O3	0.7284 (4)	0.3990 (3)	0.8858 (2)	0.0236 (8)	
O4	0.6139 (4)	0.3497 (3)	1.0325 (3)	0.0234 (8)	
O5A	0.5585 (8)	1.2043 (6)	0.4824 (4)	0.0358 (16)	0.65
O5B	0.7922 (16)	1.1019 (11)	0.3972 (9)	0.036 (3)	0.35
O6	0.9826 (6)	−0.2505 (4)	0.9067 (4)	0.0539 (13)	
O7	0.1544 (5)	0.6109 (4)	0.4944 (3)	0.0413 (11)	
O8	0.7785 (6)	0.7769 (5)	1.3297 (4)	0.075 (2)	
N1	0.5025 (5)	0.5434 (4)	0.7713 (3)	0.0192 (8)	
N2	0.7236 (5)	0.6046 (4)	1.0053 (3)	0.0205 (9)	
N3	0.2635 (6)	0.4147 (4)	0.4793 (3)	0.0273 (10)	
H3A	0.2147	0.4101	0.4278	0.033*	
H3B	0.3264	0.3518	0.5024	0.033*	
N4	0.9885 (5)	0.6171 (4)	1.3195 (3)	0.0250 (9)	
H4A	1.0185	0.6325	1.3753	0.030*	
H4B	1.0426	0.5553	1.2859	0.030*	
C1	0.7234 (6)	0.7203 (4)	0.7504 (4)	0.0206 (10)	
C2	0.7188 (6)	0.8266 (4)	0.6842 (4)	0.0222 (10)	
C3	0.8351 (7)	0.8206 (5)	0.6028 (4)	0.0259 (11)	
H3	0.9212	0.7530	0.5934	0.031*	
C4	0.8234 (7)	0.9140 (5)	0.5363 (4)	0.0299 (12)	
H4	0.9015	0.9094	0.4819	0.036*	
C5	0.6953 (8)	1.0154 (5)	0.5500 (4)	0.0325 (13)	
C6	0.5786 (7)	1.0235 (5)	0.6324 (4)	0.0339 (13)	
H6	0.4930	1.0913	0.6421	0.041*	
C7	0.5923 (7)	0.9294 (5)	0.6992 (4)	0.0286 (12)	
H7	0.5163	0.9346	0.7548	0.034*	
C8	0.6975 (5)	0.3243 (4)	0.9504 (3)	0.0172 (9)	
C9	0.7701 (6)	0.1950 (4)	0.9257 (4)	0.0217 (10)	
C10	0.7451 (7)	0.1082 (5)	0.9932 (4)	0.0269 (11)	
H10	0.6821	0.1294	1.0539	0.032*	
C11	0.8139 (7)	−0.0111 (5)	0.9706 (5)	0.0319 (12)	
H11	0.7989	−0.0695	1.0168	0.038*	
C12	0.9055 (7)	−0.0432 (5)	0.8787 (5)	0.0330 (13)	
C13	0.9269 (7)	0.0436 (5)	0.8116 (4)	0.0322 (13)	
H13	0.9874	0.0222	0.7502	0.039*	
C14	0.8600 (7)	0.1626 (5)	0.8333 (4)	0.0283 (12)	
H14	0.8749	0.2207	0.7866	0.034*	
C15	0.3770 (6)	0.6306 (5)	0.7469 (4)	0.0233 (10)	
H15	0.3460	0.6994	0.7850	0.028*	
C16	0.2918 (6)	0.6235 (4)	0.6686 (4)	0.0233 (10)	
H16	0.2064	0.6869	0.6537	0.028*	
C17	0.3337 (6)	0.5212 (4)	0.6115 (3)	0.0211 (10)	
C18	0.4627 (7)	0.4294 (5)	0.6364 (4)	0.0259 (11)	
H18	0.4936	0.3591	0.6002	0.031*	
C19	0.5449 (6)	0.4434 (4)	0.7153 (4)	0.0223 (10)	
H19	0.6324	0.3820	0.7308	0.027*	
C20	0.6678 (6)	0.7138 (4)	1.0458 (4)	0.0202 (10)	
H20	0.5980	0.7726	1.0117	0.024*	
C21	0.7102 (6)	0.7416 (5)	1.1354 (4)	0.0237 (11)	
H21	0.6680	0.8175	1.1618	0.028*	
C22	0.8170 (6)	0.6553 (5)	1.1866 (4)	0.0215 (10)	
C23	0.8753 (6)	0.5437 (4)	1.1446 (4)	0.0206 (10)	
H23	0.9476	0.4840	1.1763	0.025*	
C24	0.8253 (6)	0.5224 (4)	1.0555 (4)	0.0204 (10)	
H24	0.8643	0.4466	1.0285	0.024*	
C25	0.6803 (9)	1.1107 (6)	0.4732 (4)	0.0406 (15)	
H25A	0.748 (11)	1.094 (8)	0.410 (4)	0.035*	0.65
H25B	0.622 (17)	1.188 (4)	0.500 (10)	0.035*	0.35
C26	0.9852 (8)	−0.1697 (6)	0.8536 (5)	0.0397 (15)	
H26	1.042 (5)	−0.189 (4)	0.793 (2)	0.016 (14)*	
C27	0.2415 (6)	0.5188 (5)	0.5231 (4)	0.0241 (11)	
C28	0.8602 (7)	0.6880 (5)	1.2856 (4)	0.0268 (11)	

### Atomic displacement parameters (Å^2^)

**Table:**

	*U*^11^	*U*^22^	*U*^33^	*U*^12^	*U*^13^	*U*^23^
Cu1	0.0193 (4)	0.0219 (3)	0.0121 (3)	−0.0046 (2)	0.0078 (2)	−0.0045 (2)
O1	0.029 (2)	0.0195 (16)	0.0144 (17)	−0.0054 (15)	0.0067 (14)	−0.0028 (13)
O2	0.025 (2)	0.0264 (17)	0.0173 (18)	−0.0027 (15)	0.0065 (15)	−0.0011 (14)
O3	0.033 (2)	0.0251 (17)	0.0121 (17)	−0.0100 (16)	0.0039 (15)	−0.0005 (14)
O4	0.0201 (19)	0.0293 (18)	0.0181 (18)	−0.0055 (15)	0.0093 (14)	−0.0068 (14)
O5A	0.048 (4)	0.034 (3)	0.023 (3)	−0.011 (3)	0.004 (3)	0.000 (3)
O5B	0.050 (9)	0.027 (5)	0.027 (6)	−0.009 (5)	0.014 (5)	0.008 (5)
O6	0.046 (3)	0.037 (2)	0.079 (4)	−0.008 (2)	−0.008 (3)	−0.009 (2)
O7	0.046 (3)	0.038 (2)	0.033 (2)	0.006 (2)	−0.015 (2)	−0.0141 (19)
O8	0.062 (3)	0.083 (4)	0.053 (3)	0.043 (3)	−0.034 (3)	−0.053 (3)
N1	0.015 (2)	0.026 (2)	0.015 (2)	−0.0069 (16)	0.0081 (16)	−0.0036 (16)
N2	0.023 (2)	0.024 (2)	0.014 (2)	−0.0075 (17)	0.0065 (16)	−0.0044 (15)
N3	0.038 (3)	0.029 (2)	0.015 (2)	−0.008 (2)	0.0000 (19)	−0.0048 (17)
N4	0.025 (2)	0.031 (2)	0.015 (2)	−0.0040 (19)	0.0050 (18)	−0.0084 (18)
C1	0.023 (3)	0.025 (2)	0.013 (2)	−0.006 (2)	0.0030 (19)	−0.0040 (18)
C2	0.025 (3)	0.024 (2)	0.016 (2)	−0.007 (2)	0.006 (2)	−0.0051 (19)
C3	0.028 (3)	0.029 (2)	0.019 (3)	−0.007 (2)	0.006 (2)	−0.003 (2)
C4	0.039 (3)	0.033 (3)	0.018 (3)	−0.014 (2)	0.010 (2)	−0.003 (2)
C5	0.050 (4)	0.029 (3)	0.019 (3)	−0.014 (2)	0.006 (2)	0.001 (2)
C6	0.039 (4)	0.027 (3)	0.031 (3)	−0.003 (2)	0.002 (2)	0.000 (2)
C7	0.032 (3)	0.029 (2)	0.021 (3)	−0.005 (2)	0.011 (2)	−0.003 (2)
C8	0.005 (2)	0.024 (2)	0.019 (2)	−0.0007 (18)	0.0043 (18)	−0.0035 (18)
C9	0.015 (3)	0.024 (2)	0.023 (3)	−0.0026 (19)	0.006 (2)	−0.0065 (19)
C10	0.022 (3)	0.029 (2)	0.027 (3)	−0.006 (2)	0.006 (2)	−0.003 (2)
C11	0.030 (3)	0.026 (2)	0.041 (3)	−0.010 (2)	−0.006 (2)	0.004 (2)
C12	0.027 (3)	0.030 (3)	0.042 (3)	−0.005 (2)	−0.004 (3)	−0.011 (2)
C13	0.026 (3)	0.034 (3)	0.031 (3)	0.000 (2)	0.008 (2)	−0.016 (2)
C14	0.027 (3)	0.028 (3)	0.026 (3)	−0.005 (2)	0.008 (2)	−0.006 (2)
C15	0.020 (3)	0.027 (2)	0.018 (3)	−0.002 (2)	0.009 (2)	−0.007 (2)
C16	0.021 (3)	0.025 (2)	0.020 (3)	−0.002 (2)	0.006 (2)	−0.005 (2)
C17	0.022 (3)	0.029 (2)	0.011 (2)	−0.008 (2)	0.0121 (19)	−0.0041 (19)
C18	0.030 (3)	0.029 (2)	0.016 (2)	−0.007 (2)	0.010 (2)	−0.010 (2)
C19	0.022 (3)	0.024 (2)	0.016 (2)	−0.001 (2)	0.010 (2)	−0.0061 (19)
C20	0.017 (3)	0.022 (2)	0.016 (2)	−0.0008 (19)	0.0081 (19)	−0.0033 (18)
C21	0.024 (3)	0.025 (2)	0.018 (2)	−0.002 (2)	0.007 (2)	−0.0076 (19)
C22	0.019 (3)	0.030 (2)	0.013 (2)	−0.006 (2)	0.0054 (19)	−0.0025 (19)
C23	0.021 (3)	0.023 (2)	0.014 (2)	−0.0026 (19)	0.0085 (19)	−0.0035 (18)
C24	0.016 (3)	0.026 (2)	0.015 (2)	−0.0045 (19)	0.0124 (19)	−0.0042 (19)
C25	0.063 (5)	0.034 (3)	0.024 (3)	−0.013 (3)	0.002 (3)	0.001 (2)
C26	0.035 (4)	0.034 (3)	0.052 (4)	−0.009 (3)	−0.016 (3)	−0.003 (3)
C27	0.025 (3)	0.034 (3)	0.012 (2)	−0.010 (2)	0.011 (2)	−0.007 (2)
C28	0.024 (3)	0.035 (3)	0.018 (3)	−0.001 (2)	0.005 (2)	−0.010 (2)

### Geometric parameters (Å, º)

**Table:**

Cu1—O1	1.994 (3)	C7—C6	1.382 (8)
Cu1—O2	2.736 (4)	C7—H7	0.9300
Cu1—O3	1.949 (4)	C8—C9	1.506 (7)
Cu1—O4^i^	2.242 (3)	C9—C10	1.378 (7)
Cu1—N1	2.033 (4)	C9—C14	1.395 (7)
Cu1—N2	2.013 (4)	C10—H10	0.9300
O1—C1	1.271 (6)	C11—C10	1.390 (7)
O2—C1	1.259 (6)	C11—C12	1.395 (8)
O3—C8	1.264 (6)	C11—H11	0.9300
O4—C8	1.248 (6)	C12—C13	1.366 (9)
O4—Cu1^i^	2.242 (3)	C13—H13	0.9300
O5A—C25	1.299 (9)	C14—C13	1.384 (7)
O5A—H25B	0.61 (19)	C14—H14	0.9300
O5B—C25	1.311 (11)	C15—C16	1.367 (7)
O5B—H25A	0.43 (10)	C15—H15	0.9300
O6—C26	1.165 (8)	C16—H16	0.9300
O7—C27	1.215 (7)	C17—C16	1.385 (6)
N1—C15	1.338 (7)	C17—C27	1.508 (7)
N1—C19	1.356 (6)	C18—C17	1.388 (8)
N2—C20	1.349 (6)	C18—H18	0.9300
N2—C24	1.336 (7)	C19—C18	1.379 (7)
N3—C27	1.328 (6)	C19—H19	0.9300
N3—H3A	0.8600	C20—C21	1.371 (7)
N3—H3B	0.8600	C20—H20	0.9300
N4—C28	1.312 (7)	C21—C22	1.392 (7)
N4—H4A	0.8600	C21—H21	0.9300
N4—H4B	0.8600	C22—C28	1.511 (7)
C1—C2	1.494 (7)	C23—C22	1.381 (7)
C3—C4	1.374 (7)	C23—C24	1.370 (7)
C3—C2	1.389 (7)	C23—H23	0.9300
C3—H3	0.9300	C24—H24	0.9300
C4—H4	0.9300	C25—H25A	0.97 (2)
C5—C4	1.391 (8)	C25—H25B	0.97 (2)
C5—C25	1.479 (8)	C26—C12	1.487 (8)
C6—C5	1.398 (8)	C26—H26	0.90 (2)
C6—H6	0.9300	C28—O8	1.220 (6)
C7—C2	1.395 (7)		
			
O1—Cu1—O4^i^	87.34 (13)	C12—C11—H11	120.0
O1—Cu1—N1	88.89 (15)	C11—C12—C26	121.4 (6)
O1—Cu1—N2	90.93 (15)	C13—C12—C11	119.4 (5)
O3—Cu1—O1	150.35 (14)	C13—C12—C26	119.2 (5)
O3—Cu1—O4^i^	121.93 (13)	C12—C13—C14	121.2 (5)
O3—Cu1—N1	88.74 (16)	C12—C13—H13	119.4
O3—Cu1—N2	95.15 (16)	C14—C13—H13	119.4
N1—Cu1—O4^i^	85.80 (14)	C9—C14—H14	120.3
N2—Cu1—O4^i^	86.58 (15)	C13—C14—C9	119.5 (5)
N2—Cu1—N1	172.37 (16)	C13—C14—H14	120.3
C1—O1—Cu1	108.3 (3)	N1—C15—C16	123.3 (4)
C8—O3—Cu1	127.1 (3)	N1—C15—H15	118.4
C8—O4—Cu1^i^	148.5 (3)	C16—C15—H15	118.4
C15—N1—Cu1	119.3 (3)	C15—C16—C17	119.6 (5)
C15—N1—C19	117.5 (4)	C15—C16—H16	120.2
C19—N1—Cu1	123.1 (4)	C17—C16—H16	120.2
C20—N2—Cu1	118.3 (4)	C16—C17—C18	117.9 (5)
C24—N2—Cu1	123.9 (3)	C16—C17—C27	118.0 (5)
C24—N2—C20	117.3 (4)	C18—C17—C27	124.1 (4)
C27—N3—H3A	120.0	C17—C18—H18	120.3
C27—N3—H3B	120.0	C19—C18—C17	119.4 (4)
H3A—N3—H3B	120.0	C19—C18—H18	120.3
C28—N4—H4A	120.0	N1—C19—C18	122.3 (5)
C28—N4—H4B	120.0	N1—C19—H19	118.8
H4A—N4—H4B	120.0	C18—C19—H19	118.8
O1—C1—C2	118.2 (4)	N2—C20—C21	122.5 (5)
O2—C1—O1	123.6 (5)	N2—C20—H20	118.7
O2—C1—C2	118.2 (4)	C21—C20—H20	118.7
C3—C2—C7	119.5 (5)	C20—C21—C22	119.4 (5)
C3—C2—C1	119.9 (5)	C20—C21—H21	120.3
C7—C2—C1	120.5 (4)	C22—C21—H21	120.3
C2—C3—H3	119.9	C21—C22—C28	118.3 (5)
C4—C3—C2	120.2 (5)	C23—C22—C21	118.0 (5)
C4—C3—H3	119.9	C23—C22—C28	123.7 (5)
C4—C5—C6	120.2 (5)	C22—C23—H23	120.5
C4—C5—C25	118.8 (5)	C24—C23—C22	119.0 (5)
C6—C5—C25	121.0 (6)	C24—C23—H23	120.5
C3—C4—C5	120.3 (5)	N2—C24—C23	123.6 (5)
C3—C4—H4	119.9	N2—C24—H24	118.2
C5—C4—H4	119.9	C23—C24—H24	118.2
C5—C6—H6	120.5	O5A—C25—O5B	120.1 (8)
C7—C6—C5	119.0 (5)	O5A—C25—C5	119.9 (6)
C7—C6—H6	120.5	O5B—C25—C5	119.9 (8)
C2—C7—H7	119.6	O5A—C25—H25A	120 (5)
C6—C7—C2	120.8 (5)	C5—C25—H25A	118 (5)
C6—C7—H7	119.6	O5B—C25—H25B	120 (5)
O3—C8—C9	116.7 (4)	C5—C25—H25B	113 (7)
O4—C8—O3	125.1 (5)	H25A—C25—H25B	127 (8)
O4—C8—C9	118.2 (4)	O6—C26—C12	125.3 (7)
C10—C9—C8	120.2 (4)	O6—C26—H26	114 (3)
C10—C9—C14	119.8 (5)	C12—C26—H26	120 (3)
C14—C9—C8	120.0 (5)	O7—C27—N3	123.4 (5)
C9—C10—C11	120.0 (5)	O7—C27—C17	119.3 (5)
C9—C10—H10	120.0	N3—C27—C17	117.3 (5)
C11—C10—H10	120.0	O8—C28—N4	123.1 (5)
C10—C11—C12	120.1 (5)	O8—C28—C22	120.3 (5)
C10—C11—H11	120.0	N4—C28—C22	116.6 (4)
			
O3—Cu1—O1—C1	9.8 (5)	C6—C5—C25—O5A	0.9 (9)
O4^i^—Cu1—O1—C1	−161.5 (3)	C6—C5—C25—O5B	179.1 (9)
N1—Cu1—O1—C1	−75.7 (3)	C7—C6—C5—C4	−0.4 (9)
N2—Cu1—O1—C1	111.9 (3)	C7—C6—C5—C25	176.2 (6)
O1—Cu1—O3—C8	169.2 (4)	C6—C7—C2—C1	−173.4 (5)
O4^i^—Cu1—O3—C8	−21.0 (4)	C6—C7—C2—C3	2.1 (8)
N1—Cu1—O3—C8	−105.2 (4)	C2—C7—C6—C5	−1.1 (9)
N2—Cu1—O3—C8	68.2 (4)	O3—C8—C9—C10	178.6 (5)
O1—Cu1—N1—C19	132.7 (4)	O3—C8—C9—C14	−2.8 (7)
O3—Cu1—N1—C15	165.9 (4)	O4—C8—C9—C10	−0.7 (7)
O3—Cu1—N1—C19	−17.7 (4)	O4—C8—C9—C14	177.9 (5)
O4^i^—Cu1—N1—C15	43.8 (4)	C8—C9—C10—C11	−179.2 (5)
O4^i^—Cu1—N1—C19	−139.9 (4)	C14—C9—C10—C11	2.2 (8)
O1—Cu1—N2—C20	39.5 (3)	C8—C9—C14—C13	179.6 (5)
O1—Cu1—N2—C24	−148.6 (4)	C10—C9—C14—C13	−1.7 (8)
O3—Cu1—N2—C20	−169.5 (3)	C12—C11—C10—C9	−1.4 (8)
O3—Cu1—N2—C24	2.4 (4)	C10—C11—C12—C13	0.1 (9)
O4^i^—Cu1—N2—C20	−47.7 (3)	C10—C11—C12—C26	178.0 (5)
O4^i^—Cu1—N2—C24	124.1 (4)	C11—C12—C13—C14	0.3 (9)
Cu1—O1—C1—O2	−8.7 (6)	C26—C12—C13—C14	−177.6 (5)
Cu1—O1—C1—C2	168.4 (3)	C9—C14—C13—C12	0.5 (9)
Cu1—O3—C8—O4	−11.3 (7)	N1—C15—C16—C17	1.0 (8)
Cu1—O3—C8—C9	169.5 (3)	C18—C17—C16—C15	−0.4 (7)
Cu1^i^—O4—C8—O3	65.4 (8)	C27—C17—C16—C15	−177.4 (4)
Cu1^i^—O4—C8—C9	−115.4 (6)	C16—C17—C27—O7	12.4 (7)
Cu1—N1—C15—C16	176.0 (4)	C16—C17—C27—N3	−169.0 (4)
C19—N1—C15—C16	−0.6 (7)	C18—C17—C27—O7	−164.4 (5)
Cu1—N1—C19—C18	−177.0 (4)	C18—C17—C27—N3	14.2 (7)
C15—N1—C19—C18	−0.5 (7)	C19—C18—C17—C16	−0.6 (7)
Cu1—N2—C20—C21	171.6 (4)	C19—C18—C17—C27	176.2 (4)
C24—N2—C20—C21	−0.8 (7)	N1—C19—C18—C17	1.1 (7)
Cu1—N2—C24—C23	−172.2 (3)	N2—C20—C21—C22	1.2 (7)
C20—N2—C24—C23	−0.2 (7)	C20—C21—C22—C23	−0.5 (7)
O1—C1—C2—C3	175.4 (5)	C20—C21—C22—C28	−179.4 (4)
O1—C1—C2—C7	−9.1 (7)	C21—C22—C28—O8	15.8 (8)
O2—C1—C2—C3	−7.3 (7)	C21—C22—C28—N4	−163.5 (5)
O2—C1—C2—C7	168.2 (5)	C23—C22—C28—O8	−163.0 (6)
C4—C3—C2—C1	174.0 (5)	C23—C22—C28—N4	17.6 (7)
C4—C3—C2—C7	−1.6 (8)	C24—C23—C22—C21	−0.5 (7)
C2—C3—C4—C5	0.1 (8)	C24—C23—C22—C28	178.3 (4)
C6—C5—C4—C3	0.9 (9)	C22—C23—C24—N2	0.9 (7)
C25—C5—C4—C3	−175.8 (5)	O6—C26—C12—C11	−2.0 (10)
C4—C5—C25—O5A	177.5 (6)	O6—C26—C12—C13	175.9 (6)
C4—C5—C25—O5B	−4.2 (11)		

Symmetry code: (i) −*x*+1, −*y*+1, −*z*+2.

### Hydrogen-bond geometry (Å, º)

**Table:**

*D*—H···*A*	*D*—H	H···*A*	*D*···*A*	*D*—H···*A*
N3—H3*A*···O2^ii^	0.86	2.17	2.997 (6)	160
N3—H3*B*···O5*A*^iii^	0.86	2.27	3.044 (9)	149
N4—H4*A*···O7^iv^	0.86	2.06	2.878 (6)	158
N4—H4*B*···O2^v^	0.86	2.10	2.890 (6)	152
C3—H3···O7^vi^	0.93	2.52	3.391 (7)	155
C6—H6···O8^vii^	0.93	2.44	3.336 (9)	162
C18—H18···O5*A*^iii^	0.93	2.35	3.274 (9)	169
C23—H23···O2^v^	0.93	2.54	3.432 (7)	162
C24—H24···O6^viii^	0.93	2.50	3.217 (7)	134

Symmetry codes: (ii) −*x*+1, −*y*+1, −*z*+1; (iii) *x*, *y*−1, *z*; (iv) *x*+1, *y*, *z*+1; (v) −*x*+2, −*y*+1, −*z*+2; (vi) *x*+1, *y*, *z*; (vii) −*x*+1, −*y*+2, −*z*+2; (viii) −*x*+2, −*y*, −*z*+2.
